# The use of Complementary and Alternative Medicines (CAMs) in the treatment of diabetes mellitus: is continued use safe and effective?

**DOI:** 10.1186/1475-2891-13-102

**Published:** 2014-10-21

**Authors:** Arjuna B Medagama, Ruwanthi Bandara

**Affiliations:** Department of Medicine, University of Peradeniya, Peradeniya, Sri Lanka; Professorial Medical unit, Teaching Hospital Peradeniya, Peradeniya, Sri Lanka

**Keywords:** Complementary and Alternative Medicine (CAM), Type 2 diabetes mellitus, Cinnamon (*Cinnamomum cassia, Cinnamomum zeylanicum*), Bitter gourd (*Momordica charantia*), Crepe ginger (*Costus speciosus*), Ivy gourd (*Coccinia grandis*), Fenugreek (*Trigonella foenum-graecum*)

## Abstract

**Introduction:**

Diabetes mellitus is a major cause of morbidity and mortality worldwide, with a prevalence of 347 million in 2013. Complementary and Alternative Medicines (CAM) are a group of remedies that is fast gaining acceptance among individuals. Cinnamon, Bitter gourd (*Momordica charantia*) and Fenugreek (*Trigonella foenum-graecum*) are 3 widely used CAMs used worldwide for the treatment of diabetes. Data on safety and efficacy is limited, but the consumption is wide. Crepe ginger (*Costus speciosus*) and Ivy gourd (*Coccinia grandis*) are 2 plants used widely in the Asian region for their presumed hypoglycaemic properties.

**Objective:**

In this review, we analyzed the available evidence for the 5 CAMs mentioned above in terms of in-vitro studies, animal studies sand clinical trials. We also describe the mechanisms of hypoglycaemia and safety concerns where there is available evidence.

**Results and conclusions:**

Clinical trials that studied the hypoglycaemic effects of Cinnamon, bitter gourd, fenugreek and ivy gourd showed conflicting results. Direct comparison between studies remains a challenge in view of the baseline heterogeneity of subjects, differences in substrate preparation, variable end points and poor trial design. Short durations of study and small number of subjects studied is universal. Crepe ginger has not been studied adequately in humans to draw conclusions.

In view of the high prevalence of use and safety and efficacy issues, there is an urgent need to study their hypoglycaemic and adverse effects in well-designed long-term clinical trials.

**Electronic supplementary material:**

The online version of this article (doi:10.1186/1475-2891-13-102) contains supplementary material, which is available to authorized users.

## Background

### The burden of type 2 diabetes

Diabetes mellitus is a major cause of morbidity and mortality worldwide with an increasing prevalence. The WHO estimates a prevalence of 347 million people with diabetes worldwide in 2013 [1]. The prevalence is expected to double between 2005–2030 and the greater proportion of this increase would be in the low to middle income countries of Asia, Africa and South America [2].

There is an emerging trend worldwide for patients to use complementary and alternative medications (CAM) in an attempt to improve the outcomes of their illnesses as well as to improve general well being. In addition, CAMs have gained academic, industrial and economic interest due to its high prevalence of use.

The National Centre for Complementary and Alternative Medicine of the United States defines CAM as “a group of medical and health care systems, practices and products that are not presently considered to be part of conventional medicine”. Complementary medicine is used with conventional therapy, whereas Alternative medicine is used instead of conventional medicine. These agents seem to have become an attractive option because of the lesser-perceived adverse reactions in comparison to prescription medications. CAM incorporates herbal remedies and other forms of therapy like acupuncture, faith healing, massage therapy, hypnosis and music therapy [3].

Diabetes mellitus is an illness, where a wide array of CAMs has been used with varying success. Medicinal plants worldwide still play a prominent role in human health care. During the last few years, even in the absence of good supporting evidence, the United States alone has recorded an increase of 380% in the use of herbal remedies [4]. Around 2–3.6 million people in the United States rely on complementary and alternative medicines for the treatment of their diabetes mellitus [5].

Recent estimates show that over 80% of people living in developing countries still depend on CAM for treatment health conditions [5]. Metformin, the first choice in the treatment of type 2 diabetes originated from the plant Galega offcinalis (French lilac or goats rue) and was once considered a CAM [6]. Over 400 plants and compounds have so far been evaluated for use in T2 diabetes patients and over 1200 have been claimed to be remedies for the same illness [7].

The worldwide trend for the use of CAMs in diabetes has increased with an overall prevalence ranging between 30%-57% in some studies [8]. Diabetics are 1.6 times more likely than non-diabetics to use a CAM for a host of reasons [9].

Australia and the United Kingdom records a prevalence of 46% among diabetics [10, 11].

India, a country that is steeped in tradition and boasting a rich history of healing practices records a very high use of 67% among its diabetic population. The majority of these patients (97%) used naturoapathy, which often included herbalism [3].

It is only recently that guidelines for investigation of such remedies have developed and few herbal remedies have been studied [4].

Cinnamon, garlic preparations and fenugreek and multi-vitamins are some of the popular over the counter CAMs used among diabetics [12].

This article will attempt to bridge the evidence for cellular mechanisms of action, and human studies for using some common CAMS such as Cinnamon and fenugreek and also attempt explore anti-diabetic properties of 2 herbs and the vegetable bitter gourd.

### Cinnamon

Cinnamon, one of the most widely used flavouring agents used in the food and beverage industry worldwide has also been well recognized for its medicinal properties since antiquity. Traditional Ayurvedhic medicine has used cinnamon extracts for ailments such as arthritis, diarrhea and menstrual irregularities.

To date, about 250 species of cinnamon has been identified, 4 of which are used to obtain the spice cinnamon. True or Ceylon Cinnamon (*Cinnamomum verum*) (syn *C. zeylanicum*) is a small evergreen tree native to Sri Lanka. Chinese cassia cinnamon (*Cinnamomum aromaticum*) is the other most widely available species [13].

Preparation of cinnamon involves stripping of the outer bark of the tree and letting the inner bark to dry and curl up into its customary cinnamon quills. Cinnamon is available in either its whole quill form (Cinnamon sticks) or as ground powder in the market.

At present Cinnamon is sold as both a preventative and therapeutic supplement for many ailments including, metabolic syndrome, insulin resistance, T2 diabetes, hyperlipidaemia and arthritis [14].

Cinnamon is known to have anti-diabetic properties, in addition to which, it is also perceived to have anti-oxidant, anti-inflammatory and anti-bacterial properties [15, 16].

### Mechanisms of lowering plasma glucose

A multitude of in-vitro studies have demonstrated that cinnamon increases glucose entry into cells by enhanced insulin receptor phosphorylation and translocation of the glucose transporter GLUT4 to the plasma membrane [17]. Cinnamon increases the amount of GLUT4 receptors as well as Insulin Receptor (IR) and Insulin Receptor substrate [18, 19], thereby facilitating glucose entry into cells. The active compound responsible is believed to be a water-soluble poly-phenolic compound comprising procyanidin type A polymers [20]. Another possible mechanism for its hypoglyaemic properties, is an increase in the expression of PPAR (alpha) and PPAR (gamma), thereby increasing insulin sensitivity [21].

It has also been demonstrated that Cinnamon posses an inhibitory effect on intestinal glucosidases and pancreatic amylase. Ceylon cinnamon is the most potent inhibitor of pancreatic amylase and intestinal sucrase [22]. Cinnamon also possesses the ability to increase glycogen synthesis and inhibit gluconeogenesis, by increasing the activity of Pyruvate Kinase (PK) and decreasing that of Phoshoenol pyruvate carboxy kinase (PEPCK) [23].

A human study demonstrated its ability to delay gastric emptying as well as curb the postprandial glucose surge [24].

### Evidence from clinical trails

To date several Randomized Controlled Studies exist that studied the effect of Cinnamon in type 2 adult diabetics. These studies variably studied the effect of cinnamon on glycosylated haemoglobin, fasting plasma glucose, total cholesterol, LDL cholesterol and triglycerides [25–32].

Six trials used Cinnamon Cassia, Cinnamon aromaticum or cinnamon burmanii. 1 trial did not specify the type of cinnamon used [28]. None of the trails used Cinnamon zeylanicum.

The duration of these interventions ranged from 40 days to 4 months, the patient numbers varied from 25–109 in each trial and the amount of cinnamon administered per day varied from 1 to 6 g.

Of these studies, 2 showed no significant reduction in glycaemia [26, 27], while 5 others showed reductions in fasting plasma glucose levels and or glycosylated haemoglobin [25, 28–31].

Khan et al. in 2003 studied a total of 60 patients, who were randomized to receive 1, 3, or 6 g of cinnamon daily or a placebo. The background medication consisted of sulphonyureas only. At the end of 40 days at all 3 doses cinnamon caused a significant decrease in Fasting plasma glucose (18-29%), total cholesterol (12-26%), triglycerides (23-30%) and LDL cholesterol (7-27%) [29]. This study did not evaluate the effect of the intervention on HbA1c.

Mang et al. randomized 79 Type 2 adult diabetic patients on oral hypoglycaemic treatment or diet to receive either 3 g of cinnamon aqueous extract per day or placebo, in a randomized double blind clinical trial of 4 months duration. The study demonstrated a significant decrease of 10.3% of the initial fasting plasma glucose values. However, the trial failed to demonstrate a significant lowering of HbA1C or plasma lipids [30].

Crawford studied the administration of 1g of cinnamon as an-add on to usual care of diabetes in 109 Type 2 diabetic patients. Participants were randomly allocated to either usual care with management changes by their primary care physician or usual care with management changes plus cinnamon capsules, 1 g daily for 90 days. HbA1c was drawn at baseline and 90 days and compared with intention-to-treat analysis. Patients receiving cinnamon lowered the HbA1C by 0.83% as opposed to 0.37% reduction in patients receiving usual care alone [28].

Akilen et al. in a randomized, placebo-controlled double blind clinical trial studied the effect of Cinnamon on 58, T2 adult diabetics on oral hypoglycaemics by administering 2 g of cinnamon daily over a period of 12 weeks. The patients had an HbA1C greater than 7% at recruitment. The results demonstrated a significant reduction in HbA1C from 8.22% to 7.86%. The study also demonstrated a significant reduction in blood pressure, FPG, BMI and waist circumference at 12 weeks of treatment [31].

Suppapitiporn studied the effect of Cinnamon cassia powder in 60 T2 diabetics on oral therapy consisting of Metformin or sulphonylurea in randomized, placebo-controlled single blind clinical trial. After a 12-week period, HbA1c was decreased similarly in both groups from 8.14% to 7.76% in the cinnamon group and from 8.06% to 7.87% in the placebo group. This was not found to be statistically significant [25].

Vanschoonbeek et al. studied the effect of 1.5 g of Cinnamon cassia per day on insulin sensitivity/glucose tolerance and blood lipids on 25 Type 2 post-menopausal women on oral hypoglycaemic medications over a period of 6 weeks, in a placebo controlled study. They demonstrated that cinnamon made no significant improvement in either blood glucose or lipids at the end of the study period [26].

Blevins et al. studied the effect of 1g of cinnamon cassia on blood glucose and lipids in 60 type 2 diabetic subjects in a placebo-controlled study over a 3-month duration. The trial did not show a significant improvement in either blood glucose or lipids at the end of the study [27].

### Safety

In animal studies there is no significant toxicity of Cinnamon on the liver, but the results relating to renal functions are controversial, raising the need for more studies to evaluate its effect on the kidney.

A search of the literature for RCTS using human subjects did not reveal any reported significant adverse events when Cinnamon cassia is used in doses of 1-6 g per day. However there were four instances where patients reported a rash, hives, nausea and one hypoglycaemic seizure [33].

Although short-term trials have not demonstrated any significant adverse outcomes with Cinnamon cassia use, its high coumarin content is a concern in prolonged use [34]. Coumarins are naturally occurring plant compounds with anti-coagualnt, carcinogenic and hepatotoxic properties [35]. Cinnamon cassia which was used in almost all the human trials, has a coumarin content varying between 0.31 to 6.97 g/kg, while the coumarin content of true cinnamon or Ceylon cinnamon was only 0.017 g/Kg [34].

This high coumarin content of C. cassia has led some agencies to advocate against the regular use of cassia Cinnamon as a supplement in diabetes [36]. On the other hand, the very low content of coumarins found in *Cinnamomum zeylanicum* makes it a potentially useful medication or supplement for long-term use.

## Discussion

Akilen et al. and Crawford demonstrated a significant reduction in HbA1C with Cinnamon use [28, 31]. It is interesting to note that the trials that failed to demonstrate a significant decline in HbA1C had a mean baseline value close to 7%. (range of 6.8% to 7.1%) [26, 27]. The baseline HbA1C values for the Crawford and Akilen studies were 8.28% and 8.55% respectively. Perhaps a valid explanation for these results would be that the effect of Cinnamon is minimal when glucose control is closer to normal and that it exerts a significant effect in reducing glucose as the values increase.

Similarly Khan et al. in 2003 in their study, which lasted only 40 days was able to demonstrate a significant reduction in FPG. The HbA1C was not measured in this study. The baseline FPG was 13 mmol/l and 12 mmol/l in the placebo and treatment groups respectively and the study demonstrated the ability of cinnamon to reduce glucose in the presence of higher baseline values.

However, when the effect of cinnamon was studied in patients with impaired fasting glucose, 2 contrasting doses of 10 g and 500 mg of cinnamon per day respectively was able to reduce the fasting plasma glucose significantly [37, 38]. In both these studies, Cinnamon was used as the sole agent that had an impact on blood glucose, in contrast to the RCTS which used cinnamon as an add-on to background hypoglycaemic therapy. This raises the possibility that the glucose lowering potential of cinnamon may have been masked in the RCTS when it was used in conjunction with other medications as opposed to being used alone.

The negative outcome of the trial performed by Vanschoonbeek may be attributable to the short duration of 6 weeks, which may have given rise to a false negative HbA1C result.

Hlebowicz demonstrated the potential of cinnamon to delay gastric emptying and concluded that the fall in postprandial plasma glucose in subjects who received cinnamon was disproportionate to the delay induced in gastric emptying [24]. This raises the possibility of a dual mechanism in managing the postprandial glucose surge of diabetic patients namely, the delayed gastric emptying and the inhibition of amylase and glucosidases.

### Momordica charantia (MC) (Bitter gourd or bitter melon)

Bitter gourd is a member of the cucerbitaceae family, a perennial climber and is characterized by warty-fruit like gourds or cucumbers. The white to green unripe fruit changes to yellow as the fruit ripens. The fruit has a characteristic bitter taste, which becomes more pronounced as the fruit ripens. The fruit, as well as the whole plant is believed to posses anti-diabetic, anti-viral, anti-bacterial and anticancer properties and has been scientifically evaluated in the recent past [39].

### Mechanisms of blood glucose lowering

Results of animal and human studies show that the fruits, leaves and seed extracts of this plant possesses hypoglycaemic effects. The active compound of MC is believed to be charantin, vicine and polypeptide p. Extracts of bitter gourd is known to bear structural similarities to animal insulin [40]. Studies have demonstrated the ability MC extract to increase cellular glucose uptake by enhancing cellular insulin signaling pathways through the up regulation of GLUT4 and PI3K, as well as up regulating PPAR gamma [41].

MC preserves islet beta cells and has shown to stimulate glycogen storage by liver and insulin secretion by islets of Langerhans [42, 43].

### Evidence from clinical trials

We describe 3 randomized, double-blind controlled trials that studied the hypoglycaemic property of MC with different preparations.

John et al. randomized 50 type 2 patients on background oral hypoglycaemic therapy to receive 6 g of dried MC a day or riboflavin as placebo, over a period of 4 weeks. FPG, PPG and fructosamine levels were measured at baseline, 2 and 4 weeks thereafter. The results did not show a significant improvement in the measured parameters at the end of the study [44].

Dans et al. in 2004 studied the effect of addition of commercially available MC capsule on glycaemic control in a group of poorly controlled type 2 diabetics. The patients had an HbA1C of 7-9% at baseline and were on background OHG therapy. The patients were randomized to 2 capsules of MC 3 times a day or placebo for a duration of 3 months. The trial demonstrated a mean reduction in HbA1C of 0.217%, which was not statistically significant (p = 0.483) [45].

Fuangchan et al. in 2007 compared 3 incremental doses (500 mg, 1000 mg and 2000 mg a day) of MC against a standard dose of 1 g of Metformin in newly diagnosed, treatment naïve type 2 diabetics over a period of 4 weeks. They utilized capsules of MC containing 500 mg of dried fruit pulp. There was a significant decrease in the fructosamine levels of patients receiving metformin and those on MC 2000 mg per day. However, there was no significant lowering of the FPG or the 2-h PPG in the MC group, in spite of the lowering of the fructosamine [46].

Ahmed et al. in a single point intervention studied the effect of freshly blended MC fruit on the glycaemic effect on type 2 diabetes patients. 100 patients were studied and they were instructed to stop their pre-existing medications 3 days, prior to the intervention as a washout. All subjects then ingested freshly blended MC according to body weight. There was a statically significant reduction of 18% in PPG and FPG following the intervention [47].

In another case series, Welihinda et al. in 1986 administered juice from seedless fruits of MC to a set of type 2 diabetics to evaluate the glucose response to an OGTT. The OGTT was administered first without the MC extract, and then on a subsequent day with MC juice given 30 minutes before the glucose load. There was a significant reduction in the PPG levels in the patients when MC juice was administered. (89.5 + −6.6 mg/dl to 60.8 + 10.9 mg/dl at 1.5 hours and 81.2 + −5.5 mg/dl to 44 + −3.7 mg/dl at 2 hours respectively) [48].

### Safety

None of the studies found any serious adverse events with use of bitter melon. However there have been isolated reports of hypoglycaemic coma in children following the ingestion of bitter melon tea. Dans et al. reported the presence of abdominal pain and diarrhea as side effects in patients taking bitter melon [45].

## Discussion

Similar to other CAMs studied in diabetics the patients in these studies were heterogeneous and comparisons between studies difficult. More importantly, the dose, the method of preparation of MC, dosage and the primary outcome measures varied widely between studies. In the 3 RCTs described, 2 did not show a significant lowering of blood glucose [44, 45] and the third showed a reduction in fructosamine levels without a significant lowering of FPG or PPG [46]. Two studies [44, 46] used the dried fruit and one study [45] used a commercially available preparation. However when fresh juice was used in a case series [47, 48] there was significant reduction of FPG and PPG, highlighting perhaps the importance of the type of fruit and the method of preparation.

### Fenugreek

Fenugreek (*Trigonella foenum-graecum*) is a plant belonging to the family leguminosae. Fenugreek seed is often used as a spice as well as a medicine around the world. The leaves, chemical extracts and shoots of the plant have shown anti-oxidant, anti-diabetic and hypocholesterolaemic properties [49].

### Mechanisms of lowering blood glucose

Fenugreek seems to share many of its glucose lowering mechanisms with Cinnamon. It stimulates the tyrosine phosphorylation of the insulin receptor and enhances glucose uptake into cells [50]. In rodents, it has been shown to inhibit the intestinal disaccharidases as well as normalize the deranged levels of Pyruvate Kinase (PK) and phosphoenol pyruvate carboxykinase (PEPC K) enzymes [51].

### Evidence from clinical studies

Several clinical trials exist that demonstrated the efficacy of fenugreek to lower blood glucose. In 1998 Madar et al. demonstrated its ability to lower plasma glucose by addition of 15 g ground fenugreek to a 500 kcal meal. The study utilized 21 Type 2 diabetic subjects who were administered 2 meals, one with and one without fenugreek over a period of 4–7 days. In 17 of the 21 subjects studied, there was a significant decrease of postprandial blood glucose with the addition of fenugreek [52].

Gupta et al. in a placebo-controlled double blind clinical trial studied the effects of hydroalcoholic extracts of fenugreek on insulin resistance and glycaemic control in patients with newly diagnosed type 2 diabetes. In this study 25 patients were randomized to receive 1 g of fenugreek extract or a matching placebo over a period of 2 months. Patients also received either sulfonylurea or a biguanide if their glucose control was suboptimal. At the end of 2 months, there was a significant decrease of FPG in the fenugreek group from 148.0 + −44 to 119.9 + −25 mg/dl together with a significant decrease of HbA1C from 8.25 + −1.22% to 7.54 + −0.9% [53].

In 2008 Lu et al. randomized 69 type 2 diabetic patients on background oral hypoglycaemic therapy to receive Fenugreek 6 capsules 3 times a day (46 patients) or a matching placebo (23 patients) for a 12-week duration. In the treatment group the FPG was reduced from 155 + _31 mf/dl at baseline to 122_ + 25 mg/dl, PPBG from 240 + −72 mg/dl to 170 + −39 mg/dl and the HbA1C from 8.02% to 6.56%. All values were statistically significant [54].

In an interesting study, Kassaian et al. demonstrated that the method of preparation and delivery of fenugreek is probably an important factor in inducing its hypoglycaemic effect. Patients who were given fenugreek powder in water had a significant reduction in FPG compared to those who received the same dose in yoghurt. However, the study failed to demonstrate a significant reduction in HbA1C [55].

It should be borne in mind that the trials discussed here were trials that utilized small numbers of patients and the study durations were limited to no more than 8–12 weeks. Additionally, the preparations of fenugreek were different in each trial as were the methods of delivery and administration. More refined larger trials with uniformity in methods of preparation are required to study the hypoglycaemic effects of fenugreek.

### Safety

Fenugreek taken orally can cause mild gastrointestinal disturbances like diarrhea, dyspepsia, abdominal bloating and flatulence. Due to the insulin like activity of fenugreek, hypoglycaemia may result [52]. Hypokaleamia, dizziness and increased frequency of urination has been reported in healthy males ingesting fenugreek [49].

### Costus speciosus (Crepe Ginger)

This is a plant found in the Asian region and is used in native medicine for treating diabetes mellitus. In Sri Lanka it is common practice for diabetics to use *Costus speciosus* leaves as an additional dish to supplement their normal rice based meal. The leaf is shredded, mixed with condiments and grated coconut and eaten.

Use of *Costus speciosus* leaves to treat diabetes has not been reported to the best of our knowledge.

However, several studies exist that studied the effect of extracts of different parts of the plant on diabetic rats.

Bavarva et al. studied the effect of escalating doses of costus root extract on Alloxan induced diabetic rats. The doses of 300 mg/Kg and 400 mg/Kg significantly reduced the hyperglycaemia and improved the dyslipidaemia [56]. The observed hypoglycaemic effect was accompanied by an increased hepatic hexokinase activity and liver glycogen content in diabetic rats.

Eliza et al. studied the effect of costunolide (a crude extract of Costus speciosus) and Eramanthin (an extract of its rhizome) on diabetic rats. In both studies, plasma glucose was significantly (p <0.05) reduced in a dose-dependent manner when compared to the control. In addition, oral administration of costunolide and Eramanthin significantly decreased glycosylated hemoglobin (HbA1C), serum total cholesterol, triglyceride, LDL cholesterol and at the same time markedly increased plasma insulin, tissue glycogen, HDL cholesterol and serum protein. Additionally, both compounds restored the altered plasma enzyme (aspartate aminotransferase, alanine aminotrasferase, lactate dehydrogenase, alkaline phosphatase and acid phosphatase) levels to near normal [57, 58].

Normalization of the blood glucose levels in STZ induced diabetic rats in this study was attributed by the authors to the ability of Eramanthin to increase insulin levels. The observed increase in liver and muscle glycogen content is also probably secondary to the increase in plasma insulin levels.

### Safety

There is no safety data on humans. No significant toxic effects were observed in the animal studies.

## Discussion

We described several studies that utilized different extracts of the rhizome of Costus speciosus to treat diabetic rats with positive outcomes.

However, none of the published trials used the leaves of Costus speciosus for their studies and there are no studies on humans.

It is interesting to note that the leaves of a different species of Costus, (Costus pictus) is used in the Tamil Nadu region of India as a CAM. There are several studies that show favourable outcome for blood glucose lowering with this species.

Therefore there is an urgent need to study the practice of supplementation of diabetic patient meals with Costus speciosus leaves as its efficacy, safety and potential interactions with conventional medicines needs to be understood.

### Coccinia grandis: (Ivy gourd)

Ivy gourd also known, as baby watermelon is another tropical perennial creeper seen in the Indian subcontinent and used commonly in Sri Lanka as a raw or partly cooked leaf to supplement the rice based meal. It belongs to the family of cucerbitaceae and its scientific names are Coccinia grandis, Coccinic cordifolia and Coccinia indica.

The perceived anti-diabetic effects of the fruits and leaves of this plant have been studied both in animal models and humans.

### Mechanisms of blood glucose lowering

Although the mechanism of action of *Coccinia grandis* is not well understood, it is believed to be an insulin mimetic. Pectin, isolated from the fruit of *Coccinia grandis* has hypoglycaemic properties in rats [59]. It also possesses the ability to correct elevated levels of glucose-6-phosphotase and lactase dehydrogenase and thereby correct hypoglycaemia [60]. In addition there is some evidence of increased activation of peroxisome proliferator activated receptor-gamma activity by *Coccinia grnadis* [61]. Triterpenes, isolated from Coccinia spp may have the ability to reverse beta cell damage induced by Alloxan in experimental diabetic rats [59].

### Animal studies

Few animal studies are available with regard to exclusive use of Coccinia spp to evaluate glycaemic response. Most studies performed on diabetic rats had Coccinia being administered together with another agent, and hence not considered here.

Shibib et al. performed a case controlled study, in which two groups of 10 male rats each - one streptozotocin-induced diabetics and the other normal - were fed aqueous suspension of residue extracted from C. indica leaves with 60% ethanol, after 18 hours of fasting. After 90 minutes of oral administration, the blood sugar level had significantly decreased by 23% (p <0.01) and 28% (p <0.001) in the normal and diabetic rats. Level of blood-free fatty acid was depressed by 15% (p <0.01) and 25% (p <0.001) in the two groups respectively [62].

Manjula et al. used a an aqueous crude extract of the Coccinia leaves on alloxan induced diabetic rats and demonstrated a significant reduction in blood glucose and cholesterol levels when treated over a period of 21 days [63].

Krishnakumari et al. performed an animal study where Coccinia extract was given to streptozotocin induced diabetic rats with elevated liver markers. The study showed a favourable response in normalizing deranged hepatic enzymes [64].

### Studies in humans

In 1979 Khan et al. studied its anti-diabetic effect by administering tablets made out of homogenized and freeze dried leaves of coccinia indica or placebo tablets made of chlorophyll to newly diagnosed uncontrolled and untreated diabetics. The medication was administered for a period of 6 weeks and a 50 g oral glucose tolerance was performed at the beginning and end of the trial. There was a significant reduction in the Fasting plasma glucose and 1 and 2-hour values of the OGTT in the treatment group compared to the placebo group. The authors postulated a slow acting substance in the leaves to be responsible for the effect due the maximal decrease in glucose being seen around the third week of treatment [65].

Kuriyan et al. performed a double blind placebo controlled randomized trial.

In sixty newly detected type 2 adult diabetic patients needing only diet and lifestyle modifications at time of presentation. The treatment group received 1 g of spray dried aqueous alcohol extract of the aerial parts of the plant Coccinia cordifolia daily over 90 days. The trial demonstrated a significant decrease of both FPG and HbA1C in the subjects receiving Coccinia extract [59].

Munasinghe et al. conducted a double blind phase 1 clinical trial using the raw leaves of Coccinia grandis made into a fresh salad. The salad was made out of approximately 20 g of Coccinia leaves mixed with grated coconut and salt. 61 healthy volunteers received this salad as a test meal for breakfast and the controls another inert herb as the placebo. Both groups then underwent an oral glucose tolerance test using 75 g of glucose. The results showed a significant reduction in the test group of the 1 and 2-hour prandial glucose values [66].

### Safety

None of the animal or human studies reported any significant adverse events associated with the administration of Coccinia grandis.

## Discussion

The available trials are very heterogenous in their study populations, as well is in the methods of intervention. None of the human trials had patients who were on oral hypoglycaemic agents.

Khan et al. in 1979 used homogenized freeze-dried leaves of coccinia and Kuriyan et al. studied the effect of spray dried alcohol extract of the aerial parts of the plant. Munasinghe et al. however used the raw leaves in a salad to study the hypoglycaemic effect. Since in traditional terms the leaf is eaten as a whole in a raw or tempered salad its effect needs to be studied further in this context.

Bitter gourd (Additional file 1), Fenugreek (Additional file 2) and Cinnamon quills (Additional file 3) captioned as sold in the open market.

## Conclusion

The concept of combining dietary constituents to manage illnesses is ingrained in history. The limited evidence from studies on use of CAM in chronic illnesses strengthens this and highlights the patient acceptability and popularity. In traditional terms, these CAMs are often used as a dietary supplement rather than a medicinal preparation. In this context, when studies are planned the original context of use needs to be considered as the processes of preparation of CAM may alter the medicinal effects of plants.

A common deficiency of most trials has been the heterogeneous nature of the study population together with non-standardized preparations of the products studied, poor design of the studies, non-uniformity of outcomes, under powering of trials and the short durations of study.

The most widely studied CAM appears to be Cinnamon. As highlighted before, it seems to exert significant glucose lowering effect either when used alone [37, 38] or when used in patients with poorly controlled diabetes [28, 29, 31]. This finding which can probably be generalized for lesser-studied CAM as well suggests that there is significant glucose lowering of these substances which is probably masked by either background hypoglycaemics or by the subjects having near normal glucose values at base line.

The study product itself is a grave area of concern as the same plant has been studied in many instances using the whole raw plant or fruit, water or alcohol extracts, shade or oven dried preparations or commercialized products making interpretations and generalizations very difficult. Even when the raw plant or fruit is used, perhaps factors such as the degree of ripening and the age at harvesting may play key roles in determining the activity of crucial constituents.

On a more cautious note, herbal medicines constitute many hundreds to thousands of active and inactive ingredients, the effects of which are not known when used outside the plants natural use. Many trials have used large doses of naturally occurring substances, which would be many times the ordinary intake.

In most of the herbal remedies used in treating diabetes, the mechanisms of action have centered on insulin signaling pathways, GLUT-4 receptor translocation and the PPAR gamma activation. However, there is a need to explore different mechanisms of favourable metabolic effects these CAMs may harbor. There is also the need to incorporate the views of traditional medical practitioners into study designs whenever circumstances allow as some medicinal effects of plants may be dependent upon the season, time of harvest, degree of ripening, method of preparation etc., which in traditional terms is handed along generations of traditional practitioners.

## Electronic supplementary material

Additional file 1: **Bitter gourd fruit.** (JPEG 4 MB)

Additional file 2: **Fenugreek seeds.** (JPEG 9 MB)

Additional file 3: **Cinnamon quills.** (JPEG 6 MB)

## Abbreviations

CAM: Complementary and Alternative Medicine.
