# Supplementary figures and images for: The use of Complementary and Alternative Medicines (CAMs) in the treatment of diabetes mellitus: is continued use safe and effective?
Source: Nutr J. 2014 Oct 21;13:102. doi: 10.1186/1475-2891-13-102 (PMC4210501; doi:10.1186/1475-2891-13-102)

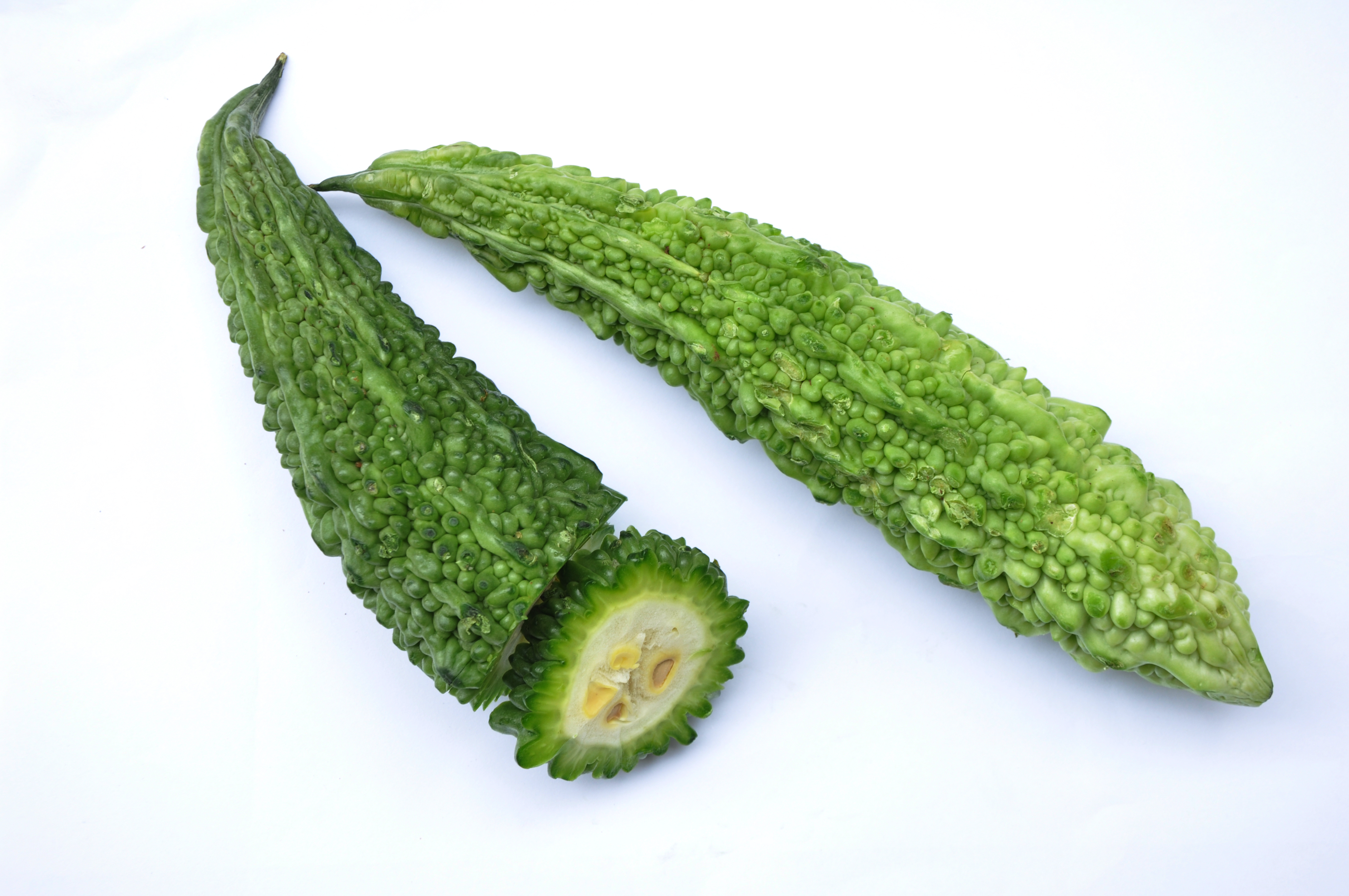

Supplement: Supplementary file 1 — Additional file 1: Bitter gourd fruit. (JPEG 4 MB) [file 12937_2014_837_MOESM1_ESM.jpeg]

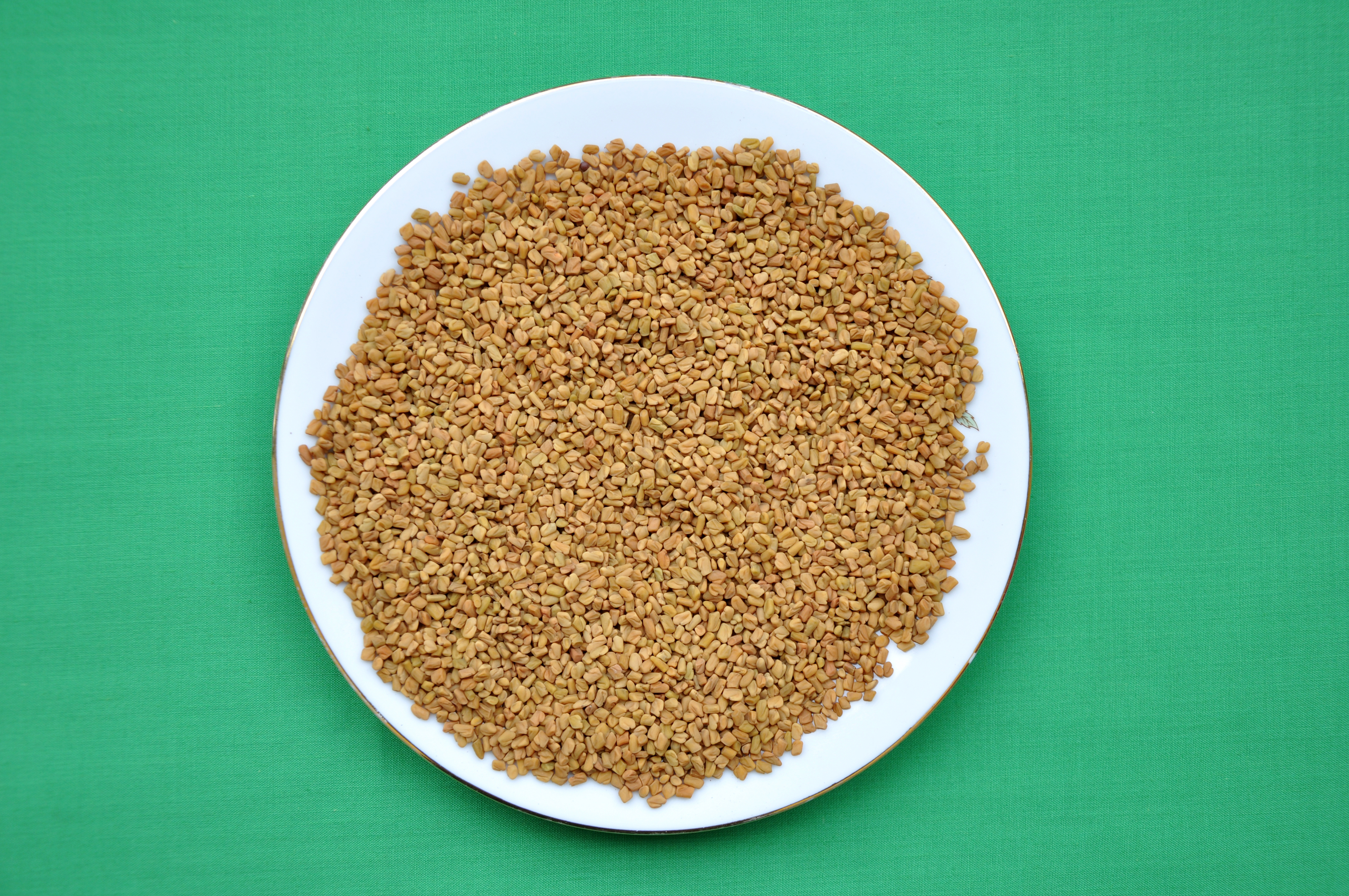

Supplement: Supplementary file 2 — Additional file 2: Fenugreek seeds. (JPEG 9 MB) [file 12937_2014_837_MOESM2_ESM.jpeg]

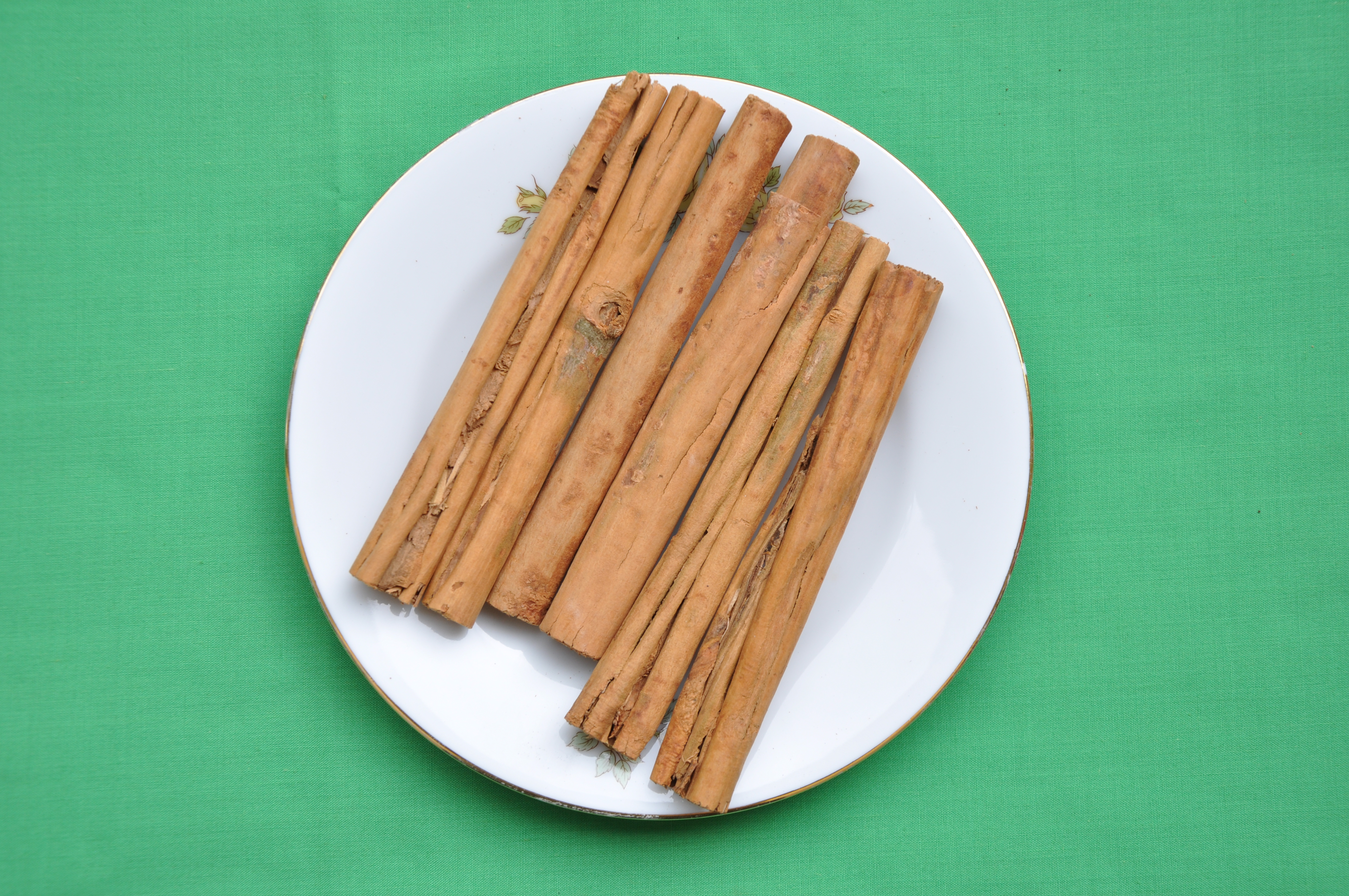

Supplement: Supplementary file 3 — Additional file 3: Cinnamon quills. (JPEG 6 MB) [file 12937_2014_837_MOESM3_ESM.jpeg]
